# Worldwide Topology of the Scientific Subject Profile: A Macro Approach in the Country Level

**DOI:** 10.1371/journal.pone.0083222

**Published:** 2013-12-09

**Authors:** Félix Moya-Anegón, Víctor Herrero-Solana

**Affiliations:** 1 CSIC/CCHS/IPP, SCImago Group, Madrid, Spain; 2 University Granada, SCImago-UGR, Granada, Spain; Brunel University, United Kingdom

## Abstract

**Background:**

Models for the production of knowledge and systems of innovation and science are key elements for characterizing a country in view of its scientific thematic profile. With regard to scientific output and publication in journals of international visibility, the countries of the world may be classified into three main groups according to their thematic bias.

**Methodology/Principal Findings:**

This paper aims to classify the countries of the world in several broad groups, described in terms of behavioural models that attempt to sum up the characteristics of their systems of knowledge and innovation. We perceive three clusters in our analysis: 1) the biomedical cluster, 2) the basic science & engineering cluster, and 3) the agricultural cluster. The countries are conceptually associated with the clusters via Principal Component Analysis (PCA), and a Multidimensional Scaling (MDS) map with all the countries is presented.

**Conclusions/Significance:**

As we have seen, insofar as scientific output and publication in journals of international visibility is concerned, the countries of the world may be classified into three main groups according to their thematic profile. These groups can be described in terms of behavioral models that attempt to sum up the characteristics of their systems of knowledge and innovation.

## Introduction

The thematic composition of the scientific output of a country is usually conditioned by factors that lie beyond the scientific system strictly speaking. We believe that the different subject profiles of countries are rooted in historical, political, economic and social terrains, and influenced by diverse issues.

No two countries are alike. However, it may be that an underlying force exerts a cluster effect on social interaction and scientific activity, affording clues for a structural analysis of output. 

It is moreover probable that the political-economic system has a strong impact on subject breakdown. If in fact this is true, countries having similar political and economic frameworks should appear near each other in classifications that make clearly distinguished categories.

Attempts by authors to differentiate countries in light of their scientific profile have tended to focus on the nature of their system of science and technology, rather than on the study of their scientific output. Yet models for the production of knowledge and systems of innovation and science can be used to characterize a country from the scientific standpoint [1]. A three-stage model describing the manner in which modern science was transmitted to the lands beyond Western Europe was developed by Basalla in the sixties [2]. Today, a great number of theoretical proposals have attempted to systematically describe and classify such models: they include Mode 2 [3], Post-Normal Science (PNS) [4], Post-Academic Science [5], Finalized Science [6,7] and the Triple-Helix model [8,9].

 All these models are *a priori* in that they focus on certain basic characteristics that are common to the different systems in existence. Therefore, it is assumed that there are different “types” of countries, established beforehand and generally responding to a dichotomous view of reality, of the sort “modern, innovative and knowledge-producing country” vs. “country with an outdated, undeveloped or poorly developed scientific system”. 

Meanwhile, there is a corpus of literature that characterizes countries a posteriori, that is, in view of empiric data of an objective nature. Deserving mention in this sense is the innovative series World Flash on Basic Research, published in Scientometrics by Schubert, Glänzel and Braun in the late 1980´s and early 1990´s. In this series, the aggregate data of the Science Citation Index are presented in summarized form, with reference to the most important countries of the world. Successive issues analyzed output, citation, collaboration, types of documents, and thematic distribution [10]. This work put forth a vast volume of data, of great interest at that point in time; yet it does not spark much debate, and advanced techniques of data analysis were not involved.

At a later date, Glänzel carried out a more detailed analysis of countries and drew up a classification in four major groupings [11]: the “western model”, a group where biomedical research prevails; the block of the former socialist countries with intense activity in chemistry and physics; the so-called “bio-environmental model” with its emphasis on biology and earth and space sciences; and finally a particular area where engineering dominates, the “Japanese model”. King arrived at a similar conclusion after studying seven of the top producers in science: UK, Germany, Japan, France, Canada, Italy and Russia [12].

Adopting this perspective in subsequent research we have authors Doré, Miquel and Okubo, among others. In perhaps the most relevant effort [13], the subject profile of 48 countries was analyzed for the period 1981-1992 by means of Correspondence Factor Analysis (CFA). CFA allows one to identify a series of factors of thematic opposition. For example, the factor φ1 encounters on the one hand chemistry, physics, and material science as opposed to clinical medicine, neurosciences, and immunology. Factor φ2 identifies agriculture in terms opposed to the geosciences and clinical medicine. Using these factors, the aforementioned authors characterized countries in a sense similar to that developed in our own work. In other studies, techniques such as cluster analysis [14], or Minimum Spanning Tree (MST) are applied, which allow for the visualization of relationships of collaboration in a schematic way [15].

Whereas the aforementioned research papers try to explain the scientific panorama working with all the foremost countries at the same time, there is also a body of work in which thematic identification is proposed for a more specific group of countries. Thus, El Alami [16] describes nine countries of the Arab World in light of eight major thematic groups. Vinkler [17] compares the scientific research structure of Western Europe with that of the countries in Central and Eastern Europe. With respect to the latter, plus the Republics of the former Soviet Union, interesting work is done by Kozlowski et al. [18]: therein, the authors analyze to what extent the communist model of scientific production remains in vigour one decade after the fall of the Berlin Wall. Jumping over to the so-called “Third World”, a selection of countries from different continents is thematically analyzed by Osareh and Wilson [19]. Finally, Okubo et al. refocus their work, this time on the Southeast Asian countries [20]. 

### Countries and fields

Each one of the countries of the world that has substantial domestic development, and some degree of impact beyond its borders, possesses moreover a system of generating technical and scientific knowledge. The question that we address in this work is whether there are great differences in the thematic specialization of their respective scientific output. 

 This approach implies the understanding that a worldwide system of scientific knowledge does indeed exist. Accordingly, the system is made up of specialized channels that are acknowledged as legitimate, and there is consensus as to their capacity to represent or characterize the world of scientific knowledge. The vast databases of Scopus (scopus.com) and WOS (isiknowledge.com) are the tools geared to control these channels, which largely take the form of prestigious scientific journals.

To gather some idea of the general thematic composition of these databases (and therefore a reflection of the worldwide system), Table 1 shows the composition of the 27 major subject areas considered by Scopus, obtained through the portal of the SCImago Journal Rank (SJR) (*scimagojr.com*). We can see that nearly a third of these correspond to the field of medicine. Far behind follow engineering, biochemistry, genetics and molecular biology and physics, each with over 10%. The rest of the areas present lower values.

**Table 1:** Thematic breakdown of World science (SJR).

Agricultural and Biological Sciences	7.0%	Heart and Planetary Sciences	4.6%	Medicine	28.6%
Arts and Humanities	0.4%	Economics, Econometrics and Finance	1.0%	Multidisciplinary	1.1%
Biochemistry, Genetics and Molecular Biology	12.8%	Energy	1.9%	Neuroscience	3.1%
Business, Management and Accounting	1.9%	Engineering	16.2%	Nursing	1.2%
Chemical Engineering	4.4%	Environmental Science	4.1%	Pharmacology, Toxicology and Pharmaceutics	4.0%
Chemistry	7.4%	Health Professions	1.6%	Physics and Astronomy	11.0%
Computer Science	4.6%	Immunology and Microbiology	3.7%	Psychology	2.0%
Decision Sciences	0.5%	Materials Science	7.4%	Social Sciences	4.1%
Dentistry	0.5%	Mathematics	3.7%	Veterinary	1.0%

Table with 27 major subject areas under Scopus, obtained through the portal SCImago Journal Rank (SJR) (scimagojr.com). The values are the percentage of each area in the total world production (total=100%).

The matter of subject bias in the categorization of science in the context of bibliometrics has been addressed in previous work by Moya Anegón et al. [21], who apply a method for comparison first introduced by Braun, Glänzel and Schubert [22] using Ulrich’s and the Science Citation Index (SCI); and Archambault [23] with the Social Science Citation Index and the Arts & Humanities Citation Index. Ulrich’s Directory is clearly a worldwide point of reference for the most comprehensive information on journals published the world over. The aim of these studies is to make a comparison of one database (SCI or Scopus), examining just how balanced the coverage that it offers is, with the Ulrich directory as the “gold standard” of reference. SCI and Scopus’s coverage with respect to Ulrich’s is evaluated by taking a series of variables into account such as journal subject distribution, geographical distribution, distribution by publishers, the language of publication, and whether peer-reviewed or not. The differences found in these papers are not statistically significant, and we may therefore consider that both Scopus and the SCI offer adequate representations of world science. Upon this premise, we explore the terrain of each country on its own. There are at least three possible case scenarios:

1 Countries are thematically very similar, with only slight variations. This scenario suggests the existence of a wide and common international matrix that transcends the borders of countries in a homogeneous way.
2 All countries are different, and the differences are random or non-systematic. This possibility would imply that science is an eminently local phenomenon, and despite being a worldwide activity, it is greatly affected by the particular reality of each country.
3 Countries present differences, yet reflecting a bias that allows them to be classified into major groups. This would indicate that, while recognizing their distinctive characteristics, we also might discern a bias that will facilitate the classification of countries by major group for their further study.

## Materials and Methods

The main data source with which we work in this line of research is the aforementioned Scopus database, through the open access portal SCImago Journal & Country Rank (SJR). The period of study was from 1996 to 2006. Information from the Web of Science (WOS) was used as a control data source; it was obtained by means of the product Essential Science Indicators (ESI). 

From the SJR we extracted information regarding the top countries of the World in terms of scientific/technical output published in journals. We use whole counting (each paper of a country). A vector of 27 components was constructed for each country to reflect the major scientific areas as registered by Scopus, given in Table 1. This dataset is open access in ZENODO portal [24].

The same was done for the ESI, although in this case there were only 22 major areas. These multidimensional matrices (of 27 and 22 dimensions, respectively) could then be processed using two separate multivariate analysis techniques that would lead to their reduction and enhance their interpretation: Principal Component Analysis (PCA) and Multidimensional Scaling (MDS).

PCA is probably the most popular multivariate statistical technique, as well as the oldest one, and it is used by almost all scientific disciplines. It analyzes a data table representing observations described by several dependent variables, which are, in general, inter-correlated. Its goal is to extract the important information from the data table and to express this information as a set of new orthogonal variables called *principal components* or *factors*. PCA also represents the pattern of similarity of the observations and the variables by displaying them as points in maps [25].

Hence, PCA attempts to achieve a projection of data in which these are optimally represented by their common denominators. This means that the dimension can be reduced and the information is synthesized by establishing a number of minimal factors that explain the variability of the data. These factors are the linear combination of the original variables and, at the same time, they are independent amongst themselves. Although they are extracted automatically, they must be identified and characterized thereafter by experts in the given data source. 

Like cluster analysis, PCA is a flexible classification method; the difference stems from the fact that the former is not exclusively determinant. Rather, it allows each element to be ascribed, and weighted, to more than one factor. This feature is extremely useful for the identification of elements that may have a strong presence in more than one zone of high variance within the matrix. 

When classifying countries, these characteristics are very important. All the countries studied present scientific output in the 27 major areas, making it necessary to use a method that accounts for the bias in each and can identify and delimit the thematic emphasis. In this way, some countries with similar emphasis will be associated by the cluster effect. We believe that the use of PCA as an analytical technique is an original notion that moreover gives sound results, as we discuss in detail below. 

Finally, MDS is used in the present study to create a bidimensional graphic representation of the factors extracted by means of PCA. While the information provided by PCA is more than sufficient for developing an analysis, the presentation of countries and factors in the form of a map means added value, enhancing the analytical potential. This combination of techniques was first suggested by Ding et al. [26].

## Results

### The three main factors

The first step consists of analyzing the results of PCA. In Table 1 we see that the three principal factors alone can explain over 90% of the variance of the complete matrix. This type of result is not common for PCA, and it suggests a strong concentration in the patterns of specialization of the countries. In Table 2, the three factors appear in decreasing order of importance, along with the percentage of variance that each explains (71.3%, 14% and 6.3%).

**Table 2:** Main factors with SJR dataset.

	% total variance	cum. %
1	71.31771	71.31771
2	14.08725	85.40496
3	6.31040	91.71536

The three main factors in PCA accumulate more than 90% of variance, which means three different country subject profiles.

In order to corroborate whether the data present some type of bias unique to this data set, we used the information from the ESI as a control set. The result, as can be seen in Table 3, is quite similar to the previous case, although here the accumulation of variance is somewhat lesser. This is most likely due to the fact that ESI does not have complete information about all the countries. In many of them, an important portion of the documents lacks thematic ascription. There are some extreme cases, such as Bahrain, where the percentage of non-ascribed records is as high as 80%.

**Table 3:** Main factors with ESI dataset.

	% total variance	cum. %
1	62.68682	62.68682
2	19.74234	82.42916
3	6.71016	89.13931

The three main factors in PCA accumulate almost 90% of variance, which means three different country subject profiles.

As we mentioned in the previous section, one of the most potent features of PCA lies in its establishment of a weighted ascription of the elements (countries) to each one of the factors. Each country will have a value associated with each one of the three factors. To see how the factors affect each one of the countries, we made a ranking of the weight that each has in every one of the factors.

In Table 4, we see that the rankings for each of the factors are quite different, and that countries that have a high weight in one factor may have a low weight in the other two. In the case where a country has similar values for two or three of the factors, these values place it midway in the ranking. 

**Table 4:** Factor loadings, by country.

**Factor 1**		**Factor 2**		**Factor 3**	
**Lebanon**	**0.92467**	**Ukraine**	**0.96722**	**Costa Rica**	**0.96094**
**Turkey**	**0.89709**	**Latvia**	**0.96660**	**Philippines**	**0.95761**
**Saudi Arabia**	**0.87953**	**Romania**	**0.96640**	**Ethiopia**	**0.93510**
**Netherlands**	**0.87445**	**Lithuania**	**0.96545**	**Indonesia**	**0.90196**
**UK**	**0.86770**	**Algeria**	**0.96040**	**Kenya**	**0.90155**
**Luxembourg**	**0.85810**	**Russian Federation**	**0.95079**	**Syrian Arab Republic**	**0.87810**
**Austria**	**0.85757**	**China**	**0.90043**	**Cameroon**	**0.86019**
**Jamaica**	**0.84666**	**Korea**	**0.89865**	**Nigeria**	**0.85301**
**United States**	**0.84638**	**Slovenia**	**0.85284**	**Sri Lanka**	**0.84216**
**Italy**	**0.82231**	**Egypt**	**0.84194**	**Ghana**	**0.81729**
**Kuwait**	**0.82200**	**Georgia**	**0.83903**	**South Africa**	**0.80199**
**Sweden**	**0.82046**	**Bulgaria**	**0.82979**	Botswana	0.79712
**Belgium**	**0.81550**	**Portugal**	**0.81991**	Zimbabwe	0.79406
**Israel**	**0.81087**	**Macedonia**	**0.81333**	Tanzania	0.77682
**Thailand**	**0.80618**	**Poland**	**0.80981**	New Zealand	0.76857
**Denmark**	**0.80126**	Singapore	0.78631	Peru	0.72990
Nepal	0.79450	Iran	0.77596	Bangladesh	0.71483
Greece	0.79434	Taiwan	0.76396	Trinidad and Tobago	0.68679
Switzerland	0.79202	India	0.74815	Uruguay	0.68127
United Arab Emirates	0.78902	Slovakia	0.72311	Senegal	0.68061
Ireland	0.78898	Japan	0.70720	Uganda	0.67246
Australia	0.78805	Hungary	0.70695	Argentina	0.65098
Finland	0.78711	Malaysia	0.69412	Cote D'ivoire	0.64852
Norway	0.78073	Hong Kong	0.68831	Viet Nam	0.64625
Tunisia	0.77347	Mexico	0.68104	Puerto Rico	0.62695
Canada	0.77243	Morocco	0.67458	Colombia	0.60595
Spain	0.75675	Cyprus	0.64783	Chile	0.60518
Germany	0.74138	Czech Republic	0.63943	Venezuela	0.59047
Oman	0.73985	Puerto Rico	0.63713	Iceland	0.58990
France	0.73444	Jordan	0.62782	Nepal	0.58045
Pakistan	0.73443	Venezuela	0.61957	Estonia	0.56555
Croatia	0.73010	Estonia	0.61711	Cuba	0.56286
Iceland	0.72747	France	0.59233	Norway	0.54418
Cote D'ivoire	0.72380	Germany	0.58842	Mexico	0.53514
Trinidad and Tobago	0.69249	Brazil	0.54834	Australia	0.53131
Senegal	0.67439	Tunisia	0.51592	Pakistan	0.52730
Uganda	0.66277	Greece	0.51487	Brazil	0.50458
Peru	0.65234	Argentina	0.50996	Thailand	0.48608
Japan	0.64546	Viet Nam	0.50246	Jamaica	0.48084
Brazil	0.64052	Chile	0.49996	Denmark	0.47289
Cuba	0.63076	Switzerland	0.49887	India	0.45707
Czech Republic	0.61223	Italy	0.48718	Malaysia	0.44074
Colombia	0.58455	Colombia	0.48238	Croatia	0.43937
Zimbabwe	0.58219	Israel	0.47704	Slovakia	0.42880
Tanzania	0.57698	Spain	0.47384	Luxembourg	0.42848
New Zealand	0.57635	Belgium	0.45586	Ireland	0.41308
Hong Kong	0.57169	Ireland	0.43793	Spain	0.41299
Morocco	0.56322	Finland	0.42914	Canada	0.41129
Jordan	0.56021	Croatia	0.42795	Finland	0.40994
Uruguay	0.51873	Canada	0.42685	Czech Republic	0.40641
Hungary	0.51829	Bangladesh	0.42683	Oman	0.40323
Ghana	0.51584	Cuba	0.41924	Jordan	0.39138
Bangladesh	0.50957	Sweden	0.41437	Portugal	0.36963
South Africa	0.50733	Oman	0.40356	Sweden	0.35441
Taiwán	0.50597	Austria	0.40060	Morocco	0.35196
Malaysia	0.50399	United Arab Emirates	0.39866	Netherlands	0.34683
Chile	0.49063	Syrian Arab Republic	0.38019	UK	0.34381
Nigeria	0.48891	United States	0.37895	Belgium	0.33824
Cyprus	0.47780	Uruguay	0.36598	United States	0.32039
Venezuela	0.47627	Kuwait	0.35742	Turkey	0.31034
Argentina	0.47399	UK	0.33396	Hungary	0.30691
Poland	0.46670	Pakistan	0.33176	Tunisia	0.30319
Sri Lanka	0.46479	Saudi Arabia	0.32487	Austria	0.29656
Mexico	0.45190	Netherlands	0.32050	France	0.29192
Iran	0.43121	Denmark	0.30848	Greece	0.28949
Macedonia	0.43044	Indonesia	0.29796	United Arab Emirates	0.28922
Bulgaria	0.42895	Australia	0.27438	Switzerland	0.28768
Viet Nam	0.42416	Thailand	0.25986	Germany	0.26008
Slovenia	0.41467	South Africa	0.25029	Lebanon	0.25991
Slovakia	0.41401	Norway	0.24698	Egypt	0.25620
India	0.41397	Turkey	0.24419	Israel	0.25477
Portugal	0.40664	Sri Lanka	0.21039	Italy	0.25327
Puerto Rico	0.39810	Luxembourg	0.20508	Poland	0.25086
Singapore	0.38912	Lebanon	0.19387	Kuwait	0.22726
Cameroon	0.38879	Cameroon	0.19096	Iran	0.22445
Estonia	0.37345	New Zealand	0.18053	Bulgaria	0.22177
Korea	0.33158	Iceland	0.14003	Saudi Arabia	0.22043
Kenya	0.33150	Uganda	0.13039	Japan	0.21064
Ethiopia	0.30266	Botswana	0.12986	Slovenia	0.17974
Egypt	0.28499	Trinidad and Tobago	0.11955	Macedonia	0.11868
Indonesia	0.27314	Peru	0.10887	Lithuania	0.11202
China	0.17828	Philippines	0.09811	Ukraine	0.07541
Georgia	0.16793	Tanzania	0.09193	Cyprus	0.07453
Philippines	0.15096	Nigeria	0.08240	Romania	0.07187
Lithuania	0.13806	Costa Rica	0.07584	Taiwan	0.06906
Costa Rica	0.13630	Jamaica	0.06673	Georgia	0.05494
Latvia	0.10271	Zimbabwe	0.05558	Hong Kong	0.04482
Romania	0.07779	Kenya	0.04850	Latvia	0.04230
Syrian Arab Republic	0.06258	Nepal	0.03566	Korea	0.02964
Botswana	0.05346	Senegal	0.03392	Singapore	0.02799
Ukraine	0.04024	Ghana	0.03078	Russian Federation	0.02091
Russian Federation	0.02751	Cote D'ivoire	0.02557	China	0.01622
Algeria	0.01103	Ethiopía	0.00376	Algeria	0.00098

The columns show the factor loading of each country. Factor loadings over 0.8 are displayed with bold style. The top countries represent each factor.

The next step would be to thematically characterize each one of the three factors. To this end, we look at the subject profile of those appearing in the top part of each factor and compare it with the world average. We took the countries showing a value equal to or greater than 0.8 for each factor, and used them to construct Tables 5, 6 and 7. 

**Table 5:** Factor 1: Biomedicine.

Country		agr	art	bio	bus	chg	chm	com	dec	den	ear	eco	ene	eng	env	hth	inm	mat	mth	med	mul	neu	nur	pha	phy	psy	soc	vet
United States	US	5,5	0,3	12,2	0,9	1,7	4,0	3,1	0,4	0,4	3,4	0,9	0,8	9,1	3,2	1,7	3,4	3,2	2,4	21,4	1,0	3,2	1,1	2,9	7,2	2,4	3,4	0,7
UK	UK	5,5	0,5	10,2	1,1	1,6	4,5	2,5	0,4	0,5	3,8	1,0	0,8	7,8	3,2	1,2	3,8	3,6	2,2	23,1	0,8	3,2	1,2	2,8	7,2	2,2	4,3	1,0
Italy	IT	4,4	0,1	11,9	0,2	1,9	5,9	3,2	0,3	0,3	4,0	0,4	0,7	8,4	2,2	0,9	3,1	4,0	3,8	23,5	0,3	3,4	0,3	3,3	11,1	0,9	0,8	0,5
Netherlands	NL	5,7	0,3	10,8	0,8	2,2	4,2	2,6	0,6	0,4	3,9	1,0	0,7	7,0	3,6	1,4	4,3	3,3	2,4	23,9	0,5	3,1	0,6	2,9	7,5	2,4	2,8	1,0
Sweden	SE	6,0	0,1	12,3	0,5	2,2	4,9	2,3	0,2	0,9	3,1	0,6	0,9	7,9	4,3	1,3	4,3	4,4	1,9	21,9	0,5	3,3	0,8	2,8	8,5	1,4	2,0	0,7
Belgium	BE	6,1	0,2	10,8	0,5	2,0	5,7	3,0	0,5	0,3	2,8	0,7	0,7	7,9	3,0	1,6	4,2	4,5	3,1	22,1	0,3	2,4	0,4	3,0	9,5	1,5	1,7	1,4
Turkey	TR	6,0	0,1	7,2	0,5	3,2	5,5	2,3	0,5	1,1	2,7	0,4	1,6	7,1	3,7	1,5	1,9	4,8	2,7	32,3	0,2	2,0	0,2	3,1	5,6	0,7	1,2	2,0
Israel	IL	5,0	0,4	10,7	0,6	2,9	4,3	4,3	0,7	0,6	2,4	0,8	0,5	7,5	2,0	1,1	3,2	3,4	5,7	21,0	0,7	3,1	0,4	2,0	11,0	2,4	3,0	0,5
Denmark	DK	8,6	0,2	13,1	0,6	1,6	4,6	2,1	0,3	0,6	4,1	0,7	0,9	5,4	4,8	1,1	5,4	2,4	2,3	23,1	0,5	2,6	0,4	2,8	7,9	0,9	1,9	1,3
Austria	AT	5,1	0,1	10,9	0,6	1,7	5,4	2,9	0,4	0,3	3,5	0,6	1,1	6,8	3,1	1,6	3,5	4,9	3,2	25,3	0,4	2,9	0,4	2,5	9,6	1,1	1,3	0,8
Thailand	TH	10,1	0,1	8,3	0,7	2,8	5,3	2,6	0,4	0,8	2,0	0,4	1,5	9,8	3,9	0,7	7,0	4,4	1,2	26,0	0,6	0,7	0,6	3,7	3,2	0,3	1,9	1,0
Saudi Arabia	SA	3,8	0,1	5,7	0,5	4,0	6,0	3,1	1,0	1,0	2,7	0,2	3,6	11,7	3,0	0,8	2,0	4,0	4,1	28,7	1,0	1,5	0,3	4,0	4,8	0,2	1,3	1,0
Kuwait	KW	3,6	0,1	6,7	0,6	6,1	5,5	3,2	1,2	1,1	3,0	0,4	3,8	12,5	4,5	0,7	3,4	3,0	5,1	21,1	2,3	1,2	0,3	3,3	2,9	1,6	2,3	0,4
Jamaica	JM	11,0	0,2	7,9	0,5	0,7	7,0	0,6	0,3	0,5	6,4	1,1	0,7	3,0	4,4	0,5	3,6	1,3	1,1	37,1	0,4	0,9	0,4	2,8	2,0	0,7	5,1	0,1
**World**		7,0	0,4	12,9	2,0	4,4	7,4	4,8	0,5	0,5	4,5	1,0	1,9	16,1	4,1	1,6	3,7	7,4	3,8	28,6	1,1	3,1	1,2	3,9	11,0	2,0	4,2	1,0

**agr**: Agricultural and Biological Sciences; **art**: Arts and Humanities; **bio**: Biochemistry, Genetics and Molecular Biology; **bus**: Business, Management and Accounting; **chg**: Chemical Engineering; **chm**: Chemistry; **com**: Computer Science; **dec**: Decision Sciences; **den**: Dentistry, **ear**: Earth and Planetary Sciences; **eco**: Economics, Econometrics and Finance; **ene**: Energy; **eng**: Engineering; **env**: Environmental Science; **hth**: Health Professions; **inm**: Immunology and Microbiology; **mat**: Materials Science; **mth**: Mathematics; **med**: Medicine; **mul**: Multidisciplinary; **neu**: Neuroscience; **nur**: Nursing; **pha**: Pharmacology, Toxicology and Pharmaceutics; **phy**: Physics and Astronomy; **psy**: Psychology; **soc**: Social Sciences; **vet**: Veterinary.

**Table 6:** Factor 2: Basic Science and Engineering.

Country		agr	art	bio	bus	chg	chm	com	dec	den	ear	eco	ene	eng	env	hth	inm	mat	mth	med	mul	neu	nur	pha	phy	psy	soc	vet
China	CN	3,4	0,0	5,8	1,2	4,7	8,8	4,8	0,3	0,0	4,8	0,1	2,4	22,0	2,3	0,1	0,9	11,2	3,8	6,7	1,1	0,5	0,1	2,1	12,1	0,1	0,6	0,1
Russian Fed.	RU	3,0	0,0	7,3	0,3	3,5	12,9	1,5	0,1	0,0	7,1	0,1	1,8	11,7	1,9	0,1	1,5	11,5	4,6	2,3	0,8	0,7	0,0	1,2	25,4	0,3	0,4	0,0
Korea	KR	2,9	0,0	10,0	0,5	4,6	7,6	5,8	0,6	0,2	1,5	0,3	1,3	17,1	1,7	0,9	3,0	10,5	3,7	8,9	0,1	1,2	0,1	3,0	12,8	0,3	0,7	0,3
Poland	PL	5,4	0,1	9,6	0,5	3,6	10,2	2,2	0,4	0,0	4,3	0,1	0,7	7,8	3,2	0,3	2,1	7,8	4,2	14,0	0,1	1,7	0,1	2,8	16,3	0,2	0,7	1,5
Portugal	PT	8,0	0,1	9,8	0,6	4,5	8,6	3,9	0,6	0,1	3,3	0,6	1,1	10,7	4,0	0,4	3,2	8,0	4,3	11,0	0,2	1,5	0,1	2,4	10,6	0,6	1,4	0,4
Egypt	EG	6,5	0,1	6,4	0,5	4,2	15,1	2,3	0,5	0,3	3,1	0,1	2,6	12,6	3,3	0,2	2,2	9,8	4,0	10,1	0,3	0,4	0,0	4,3	9,2	0,1	0,6	0,9
Romania	RO	1,3	0,0	4,0	1,3	6,1	13,0	3,0	0,4	0,1	2,0	0,1	1,3	13,8	2,1	0,2	0,5	14,1	8,4	5,6	0,1	0,3	0,0	1,0	20,4	0,2	0,4	0,1
Lithuania	LT	4,7	0,0	6,7	1,9	2,7	9,6	3,9	0,6	0,1	3,0	0,7	1,3	11,9	4,3	0,3	2,2	10,2	6,4	6,3	0,1	0,7	0,1	1,5	18,1	0,3	1,4	0,9
Algeria	DZ	4,5	0,1	3,8	0,1	5,5	8,2	5,7	0,5	0,0	3,3	0,0	2,4	17,8	2,5	0,2	0,8	14,6	6,5	3,5	0,7	0,4	0,0	1,0	17,3	0,1	0,5	0,2
Latvia	LV	3,6	0,0	7,3	0,5	3,1	11,6	4,2	0,3	0,1	1,9	0,1	1,8	13,2	2,6	0,6	2,6	13,4	2,5	5,7	0,1	0,8	0,0	1,6	20,9	0,3	0,8	0,2
Macedonia	MK	3,4	0,0	10,9	1,1	2,7	17,2	3,2	0,2	0,1	1,4	0,4	1,1	14,7	1,1	0,1	0,8	7,0	4,0	13,3	0,3	0,3	0,2	3,4	11,1	0,4	1,2	0,4
**World**		7,0	0,4	12,9	2,0	4,4	7,4	4,8	0,5	0,5	4,5	1,0	1,9	16,1	4,1	1,6	3,7	7,4	3,8	28,6	1,1	3,1	1,2	3,9	11,0	2,0	4,2	1,0

**agr**: Agricultural and Biological Sciences; **art**: Arts and Humanities; **bio**: Biochemistry, Genetics and Molecular Biology; **bus**: Business, Management and Accounting; **chg**: Chemical Engineering; **chm**: Chemistry; **com**: Computer Science; **dec**: Decision Sciences; **den**: Dentistry, **ear**: Earth and Planetary Sciences; **eco**: Economics, Econometrics and Finance; **ene**: Energy; **eng**: Engineering; **env**: Environmental Science; **hth**: Health Professions; **inm**: Immunology and Microbiology; **mat**: Materials Science; **mth**: Mathematics; **med**: Medicine; **mul**: Multidisciplinary; **neu**: Neuroscience; **nur**: Nursing; **pha**: Pharmacology, Toxicology and Pharmaceutics; **phy**: Physics and Astronomy; **psy**: Psychology; **soc**: Social Sciences; **vet**: Veterinary.

**Table 7:** Factor 3: Agriculture.

Country		agr	art	bio	bus	chg	chm	com	dec	den	ear	eco	ene	eng	env	hth	inm	mat	mth	med	mul	neu	nur	pha	phy	psy	soc	vet
Nigeria	NG	19,0	0,2	9,1	0,7	1,9	4,5	1,1	0,2	0,6	4,2	1,0	1,5	3,6	7,0	0,7	5,2	2,1	1,6	18,2	1,8	0,6	1,0	5,0	1,6	0,6	4,9	2,1
Kenya	KE	24,3	0,1	12,1	0,4	0,4	2,0	0,1	0,0	0,1	3,1	1,0	0,7	1,3	8,6	0,5	12,9	0,5	0,2	16,4	1,5	0,4	0,4	2,0	0,7	0,5	4,7	5,1
Indonesia	ID	19,2	0,1	6,6	0,8	2,6	5,2	1,3	0,3	0,4	6,4	1,4	1,7	7,3	7,8	0,3	5,6	4,0	1,1	12,5	0,5	0,3	0,5	2,7	4,9	0,4	4,6	1,4
Philippines	PH	31,5	0,1	7,3	0,8	0,9	2,5	1,1	0,1	0,3	5,5	1,6	1,4	4,0	6,8	0,4	4,5	1,2	1,7	13,6	0,4	0,6	0,7	1,8	3,9	0,7	5,3	1,3
Ethiopiía	ET	26,0	0,1	9,3	0,2	0,4	4,3	0,4	0,1	0,1	5,3	2,6	0,6	1,6	7,1	0,2	8,5	0,9	0,5	16,7	0,4	0,8	0,2	1,7	1,3	0,3	3,9	6,5
Cameroon	CM	20,0	0,1	6,8	0,2	1,0	6,8	0,8	0,1	0,0	3,9	0,9	0,6	2,9	5,4	0,4	11,5	1,7	2,5	16,5	0,5	0,5	0,2	5,2	6,3	0,3	3,2	1,5
Sri Lanka	LK	17,5	0,0	6,5	1,1	1,7	5,0	1,5	0,1	1,2	4,0	0,9	2,0	5,9	9,3	0,4	5,4	3,3	1,2	17,1	0,9	0,8	0,4	2,9	4,2	0,7	4,5	1,4
Costa Rica	CR	33,1	0,1	8,9	0,8	0,2	3,9	0,4	0,1	0,2	4,4	0,6	0,7	1,8	9,0	0,4	4,3	1,7	1,2	13,6	0,5	0,8	0,3	3,9	3,0	1,1	3,2	1,8
Ghana	GH	19,1	0,1	5,2	0,7	0,7	2,5	0,4	0,1	0,2	3,5	2,7	1,3	2,5	7,4	0,7	12,7	1,9	0,2	21,8	0,5	0,3	0,7	1,9	2,2	0,6	7,9	2,1
Syrian Arab Rep	SY	26,6	0,1	7,7	0,1	2,8	8,1	1,1	0,1	1,5	6,1	0,2	4,2	5,7	5,5	0,2	1,8	3,7	1,2	9,8	0,5	0,3	0,3	0,8	8,9	0,2	1,3	1,4
**World**		7,0	0,4	12,9	2,0	4,4	7,4	4,8	0,5	0,5	4,5	1,0	1,9	16,1	4,1	1,6	3,7	7,4	3,8	28,6	1,1	3,1	1,2	3,9	11,0	2,0	4,2	1,0

**agr**: Agricultural and Biological Sciences; **art**: Arts and Humanities; **bio**: Biochemistry, Genetics and Molecular Biology; **bus**: Business, Management and Accounting; **chg**: Chemical Engineering; **chm**: Chemistry; **com**: Computer Science; **dec**: Decision Sciences; **den**: Dentistry, **ear**: Earth and Planetary Sciences; **eco**: Economics, Econometrics and Finance; **ene**: Energy; **eng**: Engineering; **env**: Environmental Science; **hth**: Health Professions; **inm**: Immunology and Microbiology; **mat**: Materials Science; **mth**: Mathematics; **med**: Medicine; **mul**: Multidisciplinary; **neu**: Neuroscience; **nur**: Nursing; **pha**: Pharmacology, Toxicology and Pharmaceutics; **phy**: Physics and Astronomy; **psy**: Psychology; **soc**: Social Sciences; **vet**: Veterinary.

#### Factor 1 (Table 5)

The table of factor 1 was built using these countries: the United States, United Kingdom, Italy, the Netherlands, Sweden, Belgium, Turkey, Israel, Denmark, Austria, Thailand, Saudi Arabia, Kuwait and Jamaica. The common denominator that appears to group these countries together is the strong presence of medicine and biomedical research, as well as a relative poor yield in physics, engineering and materials science. Although the presence of medicine is considerable in all, there are differences regarding biochemistry, genetics and molecular biology. Those countries with high percentages of output (above World average) are the US, Israel, and Western European countries. Meanwhile, Jamaica and the Asian countries show output well below the average. Some of these countries, curiously enough, also show percentages for medicine that are well above the mean. The same phenomenon is seen for neuroscience, but to a lesser extreme. 

#### Factor 2 (Table 6)

Here the table was built with the following countries: China, Russia, Korea, Poland, Portugal, Egypt, Romania, Lithuania, Algeria, Latvia, Macedonia and the former Yugoslavia. The situation here contrasts sharply with the previous case. The biomedical areas lie below the worldwide mean, in some cases far below, like Russia. Contrariwise, output in the areas of chemistry, engineering, materials science, and physics is reasonably higher. Here the behaviour seems more homogeneous than for factor 1, though certain differences stand out. For instance, there are noteworthy high values for China in engineering and for Russia in physics. 

#### Factor 3 (Table 7)

In the table for this factor, we find the following countries: Nigeria, Kenya, Indonesia, the Philippines, Ethiopia, Cameroon, Sri Lanka, Costa Rica, Ghana and Syria. Regardless of the greater or lesser yield of these countries in the subject areas mentioned above, it seems clear that the discipline showing the most homogeneity under this factor is agriculture —all have high values in comparison with the world mean percentage. There are also high levels of production in environmental science and in immunology and microbiology, areas that might be considered related to agriculture. 

On the basis of these elements, we may characterize each one of the factors. No doubt the first will be strongly related with biomedicine, the second with sciences such as physics, chemistry and engineering in general, and the third is clearly agriculture. This can be considered the thematic division *grosso modo*.

Concerning the countries identified by each factor, we must proceed with care, as characterizing a country on the basis of its scientific output is an endeavour calling for harder work and greater subjectivity than the work with factors.

For instance, within factor 1 we have two distinct groups. On the one hand are the USA, United Kingdom, Netherlands, Luxembourg, Austria, Italy, Sweden, Belgium, Denmark, and Israel, constituting the nucleus of countries perceived as “well developed”. On the other hand we have a group of comparatively less developed countries that are nonetheless wealthy countries, such as Saudi Arabia and Kuwait, along with others that are not so wealthy: Thailand, Turkey, Jamaica and Lebanon. 

For factor 2, the list is different. We find a substantial and homogeneous group of former “iron curtain” countries: Ukraine, Latvia, Romania, Lithuania, Russia, Georgia, Bulgaria and Poland. There are also countries that had communist regimes at some point in their history: the former Yugoslavia, Slovenia and Macedonia, and China. We also see Egypt, Algeria, India and Iran, countries that bore a close association with Moscow in the past, which they used to gain effective independence from their old colonial metropolis (United Kingdom and France).

There are two countries that seem to defy characterization. The first is Korea, which, regardless of its political regime, would no doubt be influenced by its great neighbour China. The other is Portugal, a very strange case indeed, as it is the only country in Western Europe that appears clearly identified under this factor, so far away from its regional peers.

Finally, for the third factor we see no clear common denominator except for the somewhat controversial tag of Third World Countries (TWC). These are countries clearly less developed than the ones specified above. In economic terms, the best placed ones are Indonesia and South Africa, in respective positions 20 and 28 of the World Bank ranking for 2007 (worldbank.org), whereas the rest are between position 40 (Nigeria) and 98 (Ghana). This is particularly significant, as we are working with the 50 countries with the highest scientific output. Accordingly, Ghana, Sri Lanka, Syria, Ethiopia, Cameroon, Kenya and Costa Rica deserve special mention for being included in the study despite their scientific ranking well below 50.

#### Bidimensional representation

**Figure 1:**
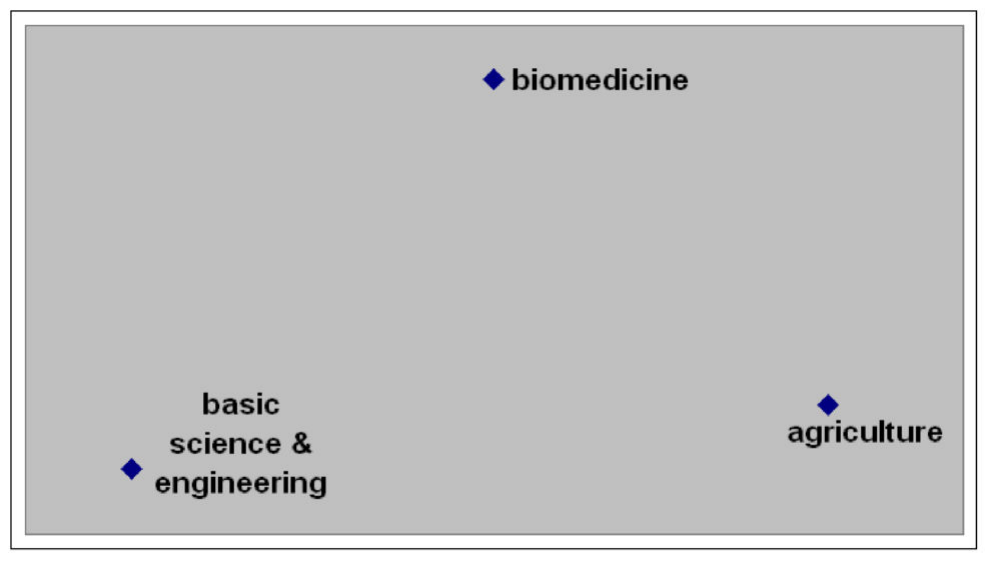
Three factors triangle. This simple MDS indicates that the factors are organized in the form of a triangle where each one of the vertices marks the pole or point of greatest affinity with the factor.

As we explained above, PCA partly characterizes each country under each factor. To fully appreciate this, we need to have some graphic depiction that reveals the relationships of all the countries with the three factors, and we can do this by means of MDS. First we shall represent only three factors (Figure 1). The map is truly a simple one, but it serves to indicate that the factors are organized in the form of a triangle where each one of the vertices marks the pole or point of greatest affinity with the factor. Because each country has relations of diverse intensity with the three factors at the same time, depending on the given intensity, each country may be represented in this triangle with the factors in its vertices. 

**Figure 2:**
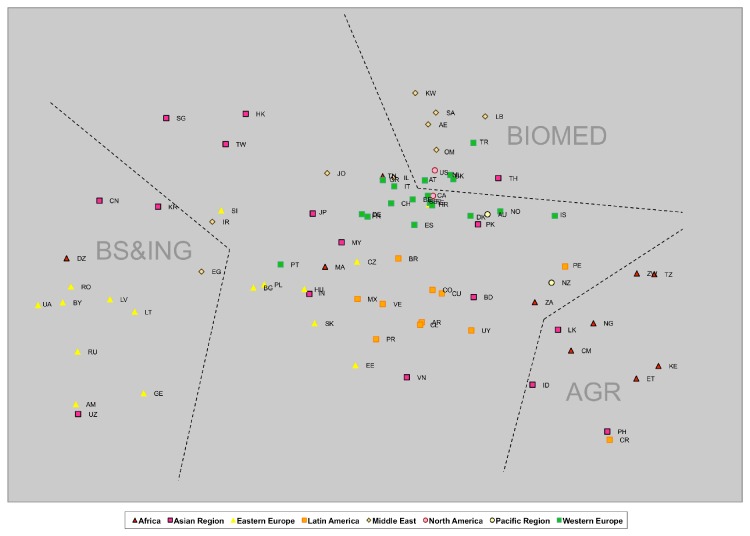
Map of countries. MDS map representing three PCA factors. Each country has relations of diverse intensity with the three factors at the same time, and may be represented in this map with the factors in its vertices.

If the map included all the countries, the representation would of course be more complex, as we see in Figure 2. Each one of the vertices is approximately indicated with tags for each factor (factor 1 – biomedicine, factor 2 – basic science & engineering, and factor 3 – agriculture). The countries are shown with their ISO code of two letters and a color that reflects their geographical region (according to the SJR portal).

Beginning on the left side of the map, we find countries sharing the greatest affinity with factor 2 (basic science & engineering). The Eastern European countries predominate, accompanied by Uzbekistan (UZ) and Algeria (DZ). A little above are the so-called “Pacific tigers”: Singapore (SG), Hong Kong (HK), Taiwan (TW), Korea (KR) and, last but not least, China (CN). Below these is a rather empty area harbouring Egypt (EG), then a group of Eastern European countries: Poland (PL), Hungary (HU), Slovakia (SK), and Slovenia (SI). Noteworthy is the intermediate position of Japan (JP), Malaysia (MY) and Portugal (PT).

Toward the right, as we approach the vertex of factor 2 (biomedicine), the number of countries increases, and appears denser. Predominant are the countries of Western Europe and North America, with their robust research in biomedicine. Above them are the countries with less output in biochemistry, genetics and molecular biology but a high yield in clinical medicine. Outstanding among these are the Middle Eastern countries: Saudi Arabia (SA), United Arab Emirates (UA), Oman (OM), Kuwait (KW), and Lebanon (LB).

Around the final pole (factor 3 – agriculture) lie mostly African countries: Kenya (KE), Ethiopia (ET), Tanzania (TZ), Zimbabwe (ZW), Nigeria (NG) and Cameroon (CM). We also see Asian countries —Philippines (PH), Indonesia (ID) and Sri Lanka (LK)— and a couple of Latin American ones —Costa Rica (CR) and Peru (PE)— in addition to the best-developed member of this group, New Zealand (NZ).

The middle area is largely populated by Latin American countries, including the three largest ones: Brazil (BR), Mexico (MX) and Argentina (AR). Alongside are the Czech Republic (CZ), Estonia (EE), Bangladesh (BD) and Vietnam (VN).

## Discussion

Many of the results described here come to reaffirm the findings of those studies cited in our Introduction. We could sum up the most noteworthy observations with the phrase “health and democracy” (assuming the risks of reductionism). There is much talk about the relationship between democratic regimes and improved life quality, especially with regards to medicine and life expectancy. This model would be the one consolidated in North America and Western Europe after WWII. In later years, the governments in question invested very substantially in the immediate medical care of the population (whose vote in electoral times could also be viewed as a matter of survival), as well as in nourishing a network of biomedical research that stands out on the horizon of traditional areas of knowledge. In this context, private enterprise dedicated to the biomedical realm gradually became a powerful sector, a “lobby” that maintains strong ties to political forces. This symbiotic relation of sorts gives rise to a development of knowledge and innovation unequalled in other countries, or in other thematic areas of scientific output. Such are the underpinnings of the schematic representation we discern for factor 1. 

Yet within the terrain of factor 1 we also have an unexpected group of Arab countries, appearing at the top. These could be referred to as the “Oil Emirates”, with Lebanon at the forefront. Although Lebanon may be a country with deep-set problems of national and political identity, the indicators of its status within the Society of Information and Knowledge put it on the par with (or even above) the wealthy Emirate states of the region [27].

One problem in the context of discussing Arabic countries is that they extend beyond the Emirate area, and could embrace countries as distant as Morocco or Iraq. Alami et al. [16] characterize this vast zone on the basis of international collaboration in eight major thematic areas, with a limited number of countries studied (including just two Emirate states: Saudi Arabia and Kuwait). The most relevant results are that Egypt is seen to have widespread collaborative efforts with several countries (Russia among them), whereas Saudi Arabia appears to secure its collaboration on either the USA or UK.

We could therefore state that the wealthy Arab countries present a model that emulates the graphic representation of the central (vs. peripheral) countries. If the key to characterizing well-developed countries resides in their investment in biomedical development, the Emirate countries would be at the lead. Yet a further distinction must be made: these countries place greater emphasis on, and invest more heavily in, clinical medicine. In contrast, the better developed countries (near the core of the graph) have more to do with biochemistry and molecular biology. This basic research calls for great effort and investment that does not translate as immediate advancement, and may be perceived as a less attractive area of research. A second consideration that serves to explain the situation of the Arab countries is their lack of attention in terms of factors 2 and 3.

With respect to factor 2, our findings would come to support the inklings of Kozlowski et al. [18], expressed at a very significant point in the history of Eastern Europe. These authors found that the post-communist countries of Central and Eastern Europe continued to maintain a Science and Technology System similar to the one that prevailed before the Wall of Berlin tumbled down. They point to a strong thematic bias, leaning toward: applied physics/condensed matters/material science; physics; physical chemistry/chemical physics; chemistry; organic chemistry/polymer science and inorganic & nuclear chemistry. Our results appear to corroborate this trend. Furthermore, the above authors underline the role of basic science, which overshadows engineering (the latter being the specialty of China and Korea).

The above authors affirm that the soviet model of science placed the bulk of its stakes on basic science for a number of reasons. Firstly, this area calls for less investment in equipment and facilities (as opposed to biomedicine). In these countries, applied research was only worthwhile or cost-effective when having directly to do with military strategy or aerospace aspirations. One of the advantages of the basic sciences is that they have very clear boundaries, and can be readily incorporated into a system founded upon classical academics. Education was more or less oriented to polishing up the prestigious reflections of the system, keeping the established scientists in a position of relative comfort and tranquility, in the vicinity of power. Hence, the soft sciences (arts and humanities), and the “human-based” research fields (social work, public health, epidemiology, etc.) tend to generate, either in the short term or the long term, situations of some conflict with respect to the establishment. The case of Soviet communism was a “scientific ideology” that proved functional in the realm of theoretical and methodological research, and was less risky than the “hot” issue-based research. Ten years after the relevant work of Kozlowski et al. [18], young post-communist democracies would appear to be incapable of defying this deeply rooted scientific/technical model. 

In a much more recent study, Vinkler [17] encounters similar conducts in a comparison of Western Europe, the USA and Japan, with the scientific situation of countries from Central and Eastern Europe (excepting the former USSR). The tendency to concentrate on life sciences in the former countries was seen to have increased (with respect to the earlier study), although the inclusion of Japan among the “Western countries” is indeed questionable. Japan, as our study and graphic display underline, has a very singular developmental posture in terms of scientific subject areas. Notwithstanding, the clues provided by Vinkler are quite useful for interpreting the position of Portugal. As we emphasized in our Results, it appears alongside Japan in the area of materials science and chemistry, an area where the countries of Eastern Europe predominate. 

The case of China and Southeast Asia is distinctive. Leydesdorff and Zhou [28] hold that China (and Iran, also factor 2) stands as a clear example of a country operating, until recently, in isolated fashion within the worldwide scientific system. While Korea, Taiwan and Singapore afford interesting case-studies because they follow the Western developmental pattern, they maintain China as a firm point of reference. A similar view is held by Okubo et al. [20], though from their standpoint China is not as supremely relevant as the “tiger” economies of Southeast Asia. The crisis at the end of the 1990´s arose in this region, and the international importance of these countries consequently declined and still lags behind in many areas of growth.

If the behavioural pattern pointed out by Leydesdorff and Zhou continues, in a near future China, Korea, Taiwan, Singapore and even Hong Kong would be further distanced from the communistic model of factor 2. This means that they might constitute an independent factor where engineering, materials science and computer science prevail (the latter particularly in Korea).

Consideration of factor 3 is more complex. In the first place, most research efforts similar to ours do not include agriculture as an independent discipline. It is sometimes linked to biology, and other times included within earth and space sciences. Such is the case of the study by Narváez Berthelemot et al. [29] about Africa, where a strong regional bias regarding agriculture is observed. 

Moreover, the countries that stand out under this factor cannot readily be perceived as a unit of any sort, neither geographical, political, ideological, nor cultural or racial. Perhaps there are economic parallels. All these countries have R+D budgets that scarcely manage to create or consolidate a multidisciplinary system of Science and Technology that might be competitive on the international level. Instead, we see an over-specialization in the area of agriculture, first and foremost. The percentage-wise figures for this factor, the highest values obtained in our study, point to a search for strong and rapid return on investment through innovation applied to the exploitation of natural resources. 

While we have no clear and consensual code of reference for these countries, they are sometimes called “Third World Countries” (TWC). This at least is the denomination used by Osareh and Wilson [19], although the criteria behind this grouping is not made explicit (e.g. India is not included in their study, but Korea is). The authors attempt to characterize this group of countries not through their output, but rather in terms of citing-cited analysis. Yet working with citation entails the great drawback of favouring TWC that are great in geographic or demographic size, while penalizing the smaller countries, such as those of the African continent. The Philippines and Kenya therefore stand out in agriculture, and Korea stands out in a subject area more appropriate for factor 2: chemistry.

Overall, what is most noteworthy in light of the results we describe is that a certain group of Latin American countries shows high citation in nearly all the thematic areas: Brazil, Mexico, Argentina and Chile. These countries are highly cited (within the realm of the TWC) by others, and also in terms of citations amongst themselves. Despite their peripheral existence, they do not rely on agricultural models or have roots in the communist model. Thus, they share a potential for developing along the lines of the USA or the central model of Science and Technology, which wields the greatest influence in the region. Deserving mention in this context is the attempt to develop integral systems that harbour all areas of knowledge, rather than merely emulating a single approach, as the wealthy “Arab Emirate model” seems to do. Notwithstanding, this sort of national scientific endeavour presents an enormous challenge for countries that dedicate less than 0.5% of their GDP to R+D [30]. This economic limitation could also explain why, in our representation, they are left somewhat isolated amid the “no man´s land” of the display. 

## Conclusions

As we have seen, in terms of scientific output and publication in journals of international visibility, the countries of the world may be classified into three main groups according to their thematic profile. These groups are reflected in behavioural models that sum up the characteristics of their systems of knowledge and innovation. We perceive three through our analysis: 

### 1): The biomedical cluster

It can be considered as characteristic of the well-developed countries, or at least of those countries with a high GDP per capita, allowing for very substantial investment in biomedical research, including research directly applied to medicine. This scientific model searches for improvement of the life quality of citizens, which is of key importance to governments not only for humanitarian reasons but also for electoral reasons, most of these governments being long-established democracies. The countries that have mature systems of Science and Technology present vigorous output in biomedical research, whereas countries that are wealthy but less developed in socio-political terms appear to invest and harvest more in clinical medicine. There appears to be a trend for wealthy countries to emulate the well-developed democracies. 

### 2): The basic science & engineering cluster

It predominates in the formerly communist countries, as the fruit of an economic and scientific society strongly state-directed, where basic research traditionally prevailed (especially in physics), along with applied research in physics and in chemistry (especially materials science). This model would appear to value scientific advancement of the country in the world ranking, with less concern for the advancement of research more directly useful for the citizens themselves.

### 3): The agricultural cluster

Here we see countries that are less developed overall, and apparently dedicate their limited resources and research efforts toward a field of more immediate yield, in view of the national natural resources. They do not possess mature scientific resources that might be directed toward biomedical or basic research. Here we identify a model that attempts to “intercept the future” by advancing in agricultural terms, including the element of livestock, while largely overlooking the need to develop an integral system for Science and Technology.

This classification resembles that proposed by Glänzel [11] quite closely, although the so-called "Japanese model" does not appear on the sidelines. However, MDS does show Japan clearly halfway between clusters 1 and 2, nearby countries as important as Singapore, Taiwan, Hong Kong and, somewhat further away, China. We believe that these countries are moving away from cluster 2, and that in the mid-term they will constitute (together with Japan) a new group where research in engineering, nanotechnology and materials science will predominate, instead of the more basic physics and chemistry. 

Finally, our analysis leads us to discern a heterogeneous group of countries, featuring a number of predominating Latin American countries, which do not clearly pertain to any of the three above models. These are largely undeveloped countries that may be aiming towards the development of an integral Science and Technology system, but lack the necessary socio-economic maturity or underlying infrastructure. They do not come under model 2 or 3. And while attempting to participate in all the areas of scientific knowledge, they do not attain the levels of the well-developed or the wealthy countries. Therefore, equal weighting of the three factors would not adequately reflect the quality of the scientific system of the country. 

The present study has focused specifically on the thematic characterization of the more productive countries in the world in terms of their scientific output, according to thematic areas acknowledged by the major databases that register publication in journals of a certain impact. This line of work will take us, in the near future, to explore:

• Analysis of the problem with respect to its evolution over time, as reflected in MDS maps. The possibility of appraising trends in output in dynamic form, year by year, also provides elements that might be lost through work on a longer 10 year basis.
• More profound ventures into the visualization of information. It would be desirable, for one, to construct a simple visual metaphor capable of reflecting a schematic visualization of international scientific/technical fluxes and refluxes, that is, a “dashboard” of countries, advancements and interchange.
• A more focused approach to the study of the smaller clusters of countries, which might reveal interesting aspects of their national scientific policies. The interpretations of the somewhat elusive countries or groups expounded in the present work are loosely based on the Economic ranking of the World Bank, a perspective that proves practical and objective. However, it would appear that politics or political history has much to do with scientific and technical evolution as well. The subjective elements that are inherent to any political analysis of a “modern country” or a “less modern country” may prove very enlightening, though they certainly entail greater risks as well.

## References

[1] Bornmann L, de Moya Anegón F, Leydesdorff L (2010) Do Scientific Advancements Lean on the Shoulders of Giants? A Bibliometric Investigation of the Ortega Hypothesis. PLOS ONE 5(10): e13327. doi:10.1371/journal.pone.0013327. PubMed: 20967252. 20967252 PMC2954151

[2] Basalla G (1967) The Spread of Western Science. Science, 156(3775): 611-622. doi:10.1126/science.156.3775.611. PubMed: 5337176. 5337176

[3] Gibbons M, Limoges C, Nowotny H, Schwartzman S, Scott P et al. (1994) The new production of knowledge: The dynamics of science and research in contemporary societies. Thousand Oaks: Sage Publications.

[4] Funtowicz S, Ravetz J (1993) Science for the post-normal age. Futures 25: 739-755. doi:10.1016/0016-3287(93)90022-L.

[5] Ziman J (2000) Postacademic science: constructing knowledge with networks and norms. In: Segerstrale U Beyond the science wars: the missing discourse about science and society. London: SUNY Press pp. 135-154.

[6] Böhme G, Van den Daele W, Krohn W (1973) Die Finalisierung der Wissenschaft. Zeitschrift für Soziologie, 2: 128-144.

[7] Schäffer W, editor (1983) Finalization in science: The social orientation of scientific progress. Dordrecht: Reidel.

[8] Etzkowitz H (2008) The Triple Helix: University-Industry-Government Innovation in Action. London: Routledge.

[9] Etzkowitz H, Leydesdorff L (1999) Whose Triple Helix? Science and Public Policy 26: 138-139.

[10] Schubert A, Glänzel W, Braun T (1989) Scientometric datafiles: A comprehensive set of indicators on 2649 journals and 96 countries in all major science fields and subfields 1981-1985. Scientometrics 16: 3-478. doi:10.1007/BF02093234.

[11] Glänzel W (2001) National characteristics in international scientific co-authorship. Scientometrics, 51: 69-115. doi:10.1023/A:1010512628145.

[12] King DA (2004) The scientific impact of nations. Nature 430: 311-316. doi:10.1038/430311a. PubMed: 15254529. 15254529

[13] Doré JC, Ojasoo T, Okubo Y, Durand T, Dudognon G, Miquel JF (1996) Correspondence factor analysis of the publication patterns of 48 countries over the period 1981-1992. Journal of the American Society for Information Science 47: 588-602. Available online at: doi:10.1002/(SICI)1097-4571(199608)47:8<588::AID-ASI3>3.0.CO;2-P

[14] Miquel JF, Ojasoo T, Okubo Y, Paul A, Doré JC (1995) World science in 18 disciplinary areas: comparative evaluation of the publication patterns of 48 countries over the period 1981-1992. Scientometrics 33: 149-167. doi:10.1007/BF02020566.

[15] Okubo Y, Miquel JF, Frigoletto L, Doré JC (1992) Structure of International collaboration in science: typology of countries through multivariate techniques using a link indicator. Scientometrics 25: 321-351. doi:10.1007/BF02028090.

[16] El Alami J, Dore C, Miquel JF (1992) International scientific collaboration in Arab countries. Scientometrics 23: 249-263. doi:10.1007/BF02020926.

[17] Vinkler P (2008) Correlation between the structure of scientific research, scientometric indicators and GDP in EU and non-EU countries. Scientometrics 74: 237-254. doi:10.1007/s11192-008-0215-z.

[18] Kozlowski J, Radosevic S, Ircha D (1999) History matters: the inherited disciplinary structure of the post-communist science in countries of Central and Eastern Europe and its restructuring. Scientometrics 45: 137-166. doi:10.1007/BF02458473.

[19] Osareh F, Wilson C (1997) Third World Countries (TWC) research publications by disciplines: a country-by-country citation analysis. Scientometrics 39: 253-266. doi:10.1007/BF02458529.

[20] Okubo Y, Doré JC, Ojasoo T, Miquel JF (1998) A multivariate analysis of publication trends in the 1980s with special reference to South-East Asia. Scientometrics 41: 273-289. doi:10.1007/BF02459045.

[21] Moya-Anegón F, Chinchilla-Rodríguez Z, Vargas-Quesada B, Corera-Álvarez E, Muñoz-Fernández et al F. (2007) Coverage analysis of Scopus: A journal metric approach. Scientometrics 73: 53-78. doi:10.1007/s11192-007-1681-4.

[22] Braun T, Glänzel W, Schubert A (2000) How balanced is the Science Citation Index´s journal coverage? A preliminary overview of macrolevel statistical data. In: Cronin B Atkins HB The web of knowledge: a festschrift in honour of Eugene Garfield. Medford: ASIS.

[23] Archambault E, Vignola-Gagne E, Côté G, Larivière V, Gingras Y (2005) Welcome to the linguistic warp zone: Benchmarking scientific output in the social sciences and humanities. In: Ingwersen P Larsen B, Proceedings of the 10th International Conference of the International Society for Scientometrics and Informetrics (ISSI). Karolinska University Press pp. 149-158.

[24] Moya-Anegón F, Herrero-Solana V (2013) Country scientific output by Scopus/SCImago major areas (1996-2006). ZENODO. doi:10.5281/zenodo.7544.

[25] Abdi H, Williams LJ (2010) Principal Component Analysis. Wiley Interdisciplinary Reviews. Computational Statistics, 2(4): 433-459. doi:10.1002/wics.101.

[26] Ding Y, Chowdhury G, Foo S (1999) Mapping the intellectual structure of information retrieval studies: an author co-citation analysis, 1987-1997. Journal of Information Science 25: 67-78. doi:10.1177/016555159902500107.

[27] Al_dwairi K, Herrero-Solana V (2007) La Sociedad de la Información en los países árabes: una aproximación al análisis de indicadores socioeconómicos. Investigación Bibliotecológica 21: 185-208.

[28] Leydesdorff L, Zhou P (2005) Are the contributions of China and Korea upsetting the world system of science? Scientometrics 63: 617-630. doi:10.1007/s11192-005-0231-1.

[29] Narváez-Berthelemot N, Russell J, Arvanitis R, Wast R, Gaillard J (2002) Science in Africa: an overview of mainstream scientific output. Scientometrics, 54: 229-241. doi:10.1023/A:1016033528117.

[30] Moya-Anegón F, Herrero-Solana V (1999) Science in America Latina: a comparison of bibliometric and scientific-technical indicators. Scientometrics 46: 299-320. doi:10.1007/BF02464780.

